# Antipsychotic Drug-Induced Increases in Peripheral Catecholamines are Associated With Glucose Intolerance

**DOI:** 10.3389/fphar.2022.765905

**Published:** 2022-02-15

**Authors:** Heidi N. Boyda, Michelle Pham, Joyce Huang, Amanzo A. Ho, Ric M. Procyshyn, Jessica W. Y Yuen, William G. Honer, Alasdair M. Barr

**Affiliations:** ^1^ Department of Anesthesiology, Pharmacology and Therapeutics, Faculty of Medicine, University of British Columbia, Vancouver, BC, Canada; ^2^ Department of Psychiatry, Faculty of Medicine, University of British Columbia, Vancouver, BC, Canada; ^3^ British Columbia Mental Health & Substance Use Services Research Institute, Vancouver, BC, Canada; ^4^ Centre for Brain Health, University of British Columbia, Vancouver, BC, Canada

**Keywords:** antipsychotic, catecholamine, norepinephrine, epinephrine, dopamine, glucose intolerance, side-effect

## Abstract

The second-generation antipsychotic drugs are widely used in the field of psychiatry, for an expanding number of different conditions. While their clinical efficacy remains indispensable, many of the drugs can cause severe metabolic side-effects, resulting in an increased risk of developing cardiometabolic disorders. The physiological basis of these side-effects remains an ongoing area of investigation. In the present study, we examined the potential role of peripheral catecholamines in antipsychotic-induced glucose intolerance. Adult female rats were acutely treated with either the first-generation antipsychotic drug haloperidol (0.1, 0.5 or 1 mg/kg) or the second-generation drugs risperidone (0.25, 1.0 or 2.5 mg/kg), olanzapine (1.5, 7.5 or 15 mg/kg) or clozapine (2, 10 or 20 mg/kg) or vehicle. Fasting glucose levels were measured and then animals were subjected to the intraperitoneal glucose tolerance test. Levels of peripheral norepinephrine, epinephrine and dopamine were concurrently measured in the same animals 75, 105 and 135 min after drug treatment. All antipsychotics caused glucose intolerance, with strongest effects by clozapine > olanzapine > risperidone > haloperidol. Plasma catecholamines were also increased by drug treatment, with greatest effects for norepinephrine and epinephrine caused by clozapine > risperidone > olanzapine > haloperidol. Importantly, there were strong and statistically significant associations between norepinephrine/epinephrine levels and glucose intolerance for all drugs. These findings confirm that increases in peripheral catecholamines co-occur in animals that exhibit antipsychotic-induced glucose intolerance, and these effects are strongly associated with each other, providing further evidence for elevated catecholamines as a substrate for antipsychotic metabolic side-effects.

## Introduction

The antipsychotic drugs remain the most effective form of pharmacotherapy for schizophrenia spectrum disorders. These compounds, which were initially developed in the 1950s (Shen, 1999; Seeman, 2021), are used widely in the field of psychiatry to treat a range of psychotic disorders, and have expanded in use beyond their original indications to include other psychiatric conditions and patient populations (Hershenberg et al., 2014; Vasudev et al., 2018; Del Casale et al., 2019; Mela et al., 2020; Ameer and Saadabadi, 2021; Lian et al., 2021). The antipsychotics are traditionally classified into at least two categories, which include the earlier, first-generation drugs, and the more recent second-generation compounds [some consider the dopamine D2 receptor partial agonists to represent a third-generation (Mailman and Murthy, 2010)]. While the second-generation drugs have significantly displaced the first-generation ones in clinical use, due to their lower incidence of neurological side-effects (Rummel-Kluge et al., 2010), the second-generation antipsychotics are associated with their own side-effect profiles, which commonly include metabolic dysregulation. These metabolic side-effects result in increased risk for developing cardiometabolic disorders with chronic use (Newcomer, 2007; Kessing et al., 2010; Deng, 2013; Tse et al., 2014; Bozymski et al., 2018; Kim et al., 2018; Yuen et al., 2018; Tumiel et al., 2019), which is a particular concern as many idiopathic psychotic disorders require lifelong treatment with these medications (Lindenmayer and Khan, 2004).

At present, the biochemical pathways that underlie the metabolic side-effects of the second-generation antipsychotics are not fully established. It appears likely that the metabolic side-effects are unrelated to the primary mechanism of clinical efficacy of these drugs (Reynolds and Kirk, 2010), which is antagonism of mesolimbic dopamine D2 receptors. Instead, a wide number of non-dopaminergic receptors and other physiological substrates are posited to play key roles in the metabolic side-effects of the second-generation antipsychotics, including both central and peripheral mechanisms (De Hert et al., 2011; Reynolds and McGowan, 2017; Bush et al., 2018; Kowalchuk et al., 2019; Marteene et al., 2019). In this regard, preclinical rodent models are especially helpful in advancing our understanding of the biochemical pathways involved, as they provide the opportunity for invasive experimental procedures, direct experimental control, and are not prone to the multifactorial causes of metabolic dysregulation observed in antipsychotic-treated patients, which include diet, exercise and non-prescribed substance use (Laursen et al., 2012; Marteene et al., 2019).

Rodent models of antipsychotic-induced side-effects reliably demonstrate metabolic changes that parallel those observed in humans, including glucose dysregulation, insulin resistance and weight gain (Baptista et al., 1999; Baptista et al., 2004; Girault et al., 2012; Weston-Green et al., 2013; Ersland et al., 2015; Babic et al., 2018; Shamshoum et al., 2019; Lian and Deng, 2020; Ashraf et al., 2021), using homologous techniques such as the glucose tolerance test (GTT) (Eyth et al., 2021). A number of these preclinical studies provide strong evidence that the peripheral catecholamines norepinephrine and epinephrine play an important role in regulating these metabolic side-effects (Savoy et al., 2010; Nagata et al., 2016; Nagata et al., 2018), and are consistent with clinical studies in antipsychotic-treated patients (Brown et al., 1997; Spivak et al., 1998; Elman et al., 1999). Given the potent control that peripheral catecholamines exert over glucose regulation and insulin release (Cryer, 1993; Barth et al., 2007), they represent a viable substrate for further, translational study. Recently, we demonstrated that acute treatment with different antipsychotic drugs caused substantial increases in levels of peripheral norepinephrine, epinephrine and dopamine, and these effects were greatest by the drugs with the highest metabolic liability (Boyda et al., 2020). However, metabolic indices were not assessed, and so the link between elevated catecholamines and metabolic dysregulation was not confirmed. Therefore, the goal of the present study was to use a preclinical model to treat rats with multiple different antipsychotic drugs, across a range of doses, and concurrently measure the effects on both peripheral catecholamine levels *and* glucose dysregulation, using the GTT. This would potentially allow us to confirm that glucose intolerance was associated with elevated catecholamine levels, as well as determine the strength of that association for each of the individual catecholamines involved.

## Materials and Methods

### Animals

Experimentally-naive, adult female Sprague-Dawley rats (225–250 g; Charles River, Montreal, QC, Canada) were habituated to the UBC animal rodent colony for 1 week before all experiments started. Our laboratory uses female rats because they exhibit consistent metabolic dysregulation following antipsychotic drug treatment (Weston-Green et al., 2010; Davey et al., 2012). Rats were group housed in large polycarbonate cages, and provided *ad libitum* access to tap water and food (Purina rat chow). Animals were maintained on a 12-h light-dark cycle (lights on at 07:00 h) in a temperature-controlled colony (22 ± 1°C) (Barr and Phillips, 2002). All experiments were completed in accordance with the National Institutes of Health Guide for the Care and Use of Laboratory Animals. The University of British Columbia’s Animal Care and Use Committee approved all procedures.

### Pharmaceutical Drugs

The choice of antipsychotic drugs represented those used commonly in clinical practice, as well as antipsychotics whose metabolic effects had been determined previously in rodent models. The range of drug doses was also based on previous studies of the metabolic side-effects of antipsychotic drugs (Boyda et al., 2013a; Boyda et al., 2021). Each drug included three doses, which spanned a 10-fold concentration range. Drug doses for the study included haloperidol (0.1, 0.5 and 1.0 mg/kg), risperidone (0.25, 1.0 and 2.5 mg/kg), olanzapine (1.5, 7.5 and 15.0 mg/kg) and clozapine (2.0, 10.0 and 20.0 mg/kg) [Toronto Research Chemicals Inc., Toronto, ON, Canada]. Drug solutions were prepared fresh daily: risperidone, olanzapine and clozapine were formulated in 50% polyethylene glycol 400, 40% distilled water and 10% ethanol; haloperidol was prepared in 0.3% tartaric acid.

### Intraperitoneal GTT and Blood Collection

**FIGURE 1:**
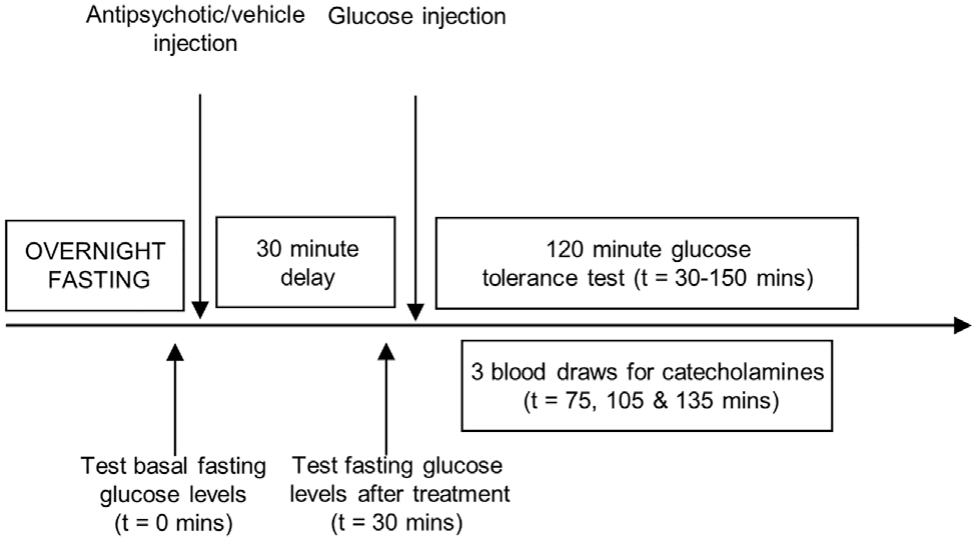
Experimental procedures. Representation of the sequence of events during the intraperitoneal glucose tolerance test. Adult, female Sprague-Dawley rats were fasted overnight. A baseline fasting blood glucose measurement was taken (*t* = 0 min), immediately followed by treatment with one of the four antipsychotic drugs or vehicle. Glucose levels were measured 30 min later (*t* = 30 min), followed by the glucose challenge (1 g/kg/ml, i.p.) with repeated sampling of blood glucose readings every 15 min for 2 hours (*t* = 30–150 min). Three larger blood draws were performed at *t* = 75, 105 and 135 min to measure peripheral catecholamine levels.

Animals were randomly assigned to treatment [*n* = 10–11 per group], and were fasted overnight (Figure 1). Blood glucose measurements were determined using a hand-held glucometer (One Touch Ultra) by wrapping the rat in a towel and procuring a drop of blood from the saphenous vein with a 25-gauge needle. After measuring baseline fasting blood glucose at *t* = 0 min, animals received treatment with a single dose of one of the four antipsychotic drugs or vehicle by subcutaneous injection, in a volume of 1 ml/kg. A second glucose measurement was taken at *t* = 30 min to assess the effect of drug treatment on fasting glucose levels. This was followed by the glucose challenge (1 g/kg/ml, i.p.), marking the start of the IGTT. Glucose readings were then repeatedly determined every 15 min for the next 2 h. Animal handlers were blinded to the treatment conditions. In addition to the IGTT, a larger volume of blood (200 µl) was collected from the saphenous vein via capillary tubes at three different time points after the start of the study (t = 75, 105 and 135 min after the baseline fasting blood glucose measurement) for measurement of peripheral plasma catecholamine levels. Samples were centrifuged (10,000 RPM, 10 min, 4°C) and stored at −80°C until analysis.

### Determination of Plasma Catecholamine Levels

Standards for norepinephrine, epinephrine and dopamine (50–25,600 pg/ml) were prepared by dilution of stock 1 mg/ml solution with diluent (ThermoFisher, Sunnyvale, CA, United States) for generation of a standard curve. The internal standard (IS) solution was 20 ng/ml 3,4-dihydroxybenzylamine (DHBA). Samples were prepared by adding 90 μl of 3 M Tris-5% EDTA buffer, 10 μl of DHBA and 50 μl of plasma/standard solution to a centrifuge tube containing 5.0 mg activated alumina oxide. Samples were vortexed and placed on a rotary mixer for 10 min at 4°C, followed by centrifugation and removal of the supernatant. Addition of 400 µl of ultra-pure water was followed by aspiration, performed three consecutive times, followed by centrifugation. 50 µl of .1 M perchloric acid was added and samples were mixed for 10 min. Samples were vortexed, centrifuged and the supernatant was injected into the HPLC system.

### High-Performance Liquid Chromatography-Electrochemical Detection

Catecholamines were analyzed by HPLC coupled to ECD. A Shimadzu series HPLC system (ESA Coulochem III electrochemical detector) separated analytes on a ESA HR-80 column (Hill et al., 2007). Mobile phase was .7% sodium phosphate, 3% sodium citrate, .02% EDTA, 0.2% diethylamine HCl, .1% 1-octanesulfonic acid, 5% acetonitrile, 0.2% dimethylacetamide and water, pH–adjusted to 3.1. A 20 µl injection was loaded at 0.4 ml/min; all reagents were HPLC-grade. The ECD system used an ESA 5020 guard cell (200 mV) and an ESA 5011 analytical cell (E_1_ = −150 mV; E_2_ = 225 mV). All samples were processed blind to treatment condition, with chromatogram peaks analyzed by LC solution.

### Statistical Analysis

Fasting blood glucose levels both prior to and following drug treatment (but prior to the glucose challenge) were analyzed by one-way analysis of variance (ANOVA), with drug dose as the between-subjects group factor. For the IGTT, glucose levels were analyzed by repeated-measures ANOVA, with drug treatment as the between-subjects group factor and time as the within-subjects measure. Glucose levels were also summed as the area-under-the-curve throughout the 120 min procedure and analyzed by one-way ANOVA, with drug treatment as the between-subjects group factor. Data obtained from the larger blood samples at [*t* = 75, 105 and 135 min] included plasma levels of norepinephrine, epinephrine and dopamine. These data were analyzed by a repeated-measures ANOVA, with drug treatment as the between-subjects group factor, and time as the within-subjects factor. Catecholamine data were also analyzed as the area-under-the-curve for the three timepoints. Main effects or interactions were followed up with the LSD post-hoc test. Correlations were conducted using the Pearson correlation coefficient (SPSS Version 24.0. Armonk, NY, United States: IBM Corp). Follow-up analyses using mediation analysis (Baron and Kenny, 1986) were conducted to determine if the concentrations of catecholamines acted as a mediator variable to moderate the effects of drug treatment (dose) on glucose intolerance in the IGTT. For these analyses, drug dose represented the independent variable, the area-under-the-curve (AUC) of glucose levels for the IGTT was the dependent variable, and the AUC of the catecholamine concentrations was the intervening/mediating variable. Unstandardized variable coefficients were calculated using bivariate and multiple linear regression, and mediator effects were analyzed with the Sobel test for significance.

## Results

### Intraperitoneal GTT

#### Data From the Intraperitoneal GTT Were Analyzed Separately for Each Antipsychotic Drug

**FIGURE 2:**
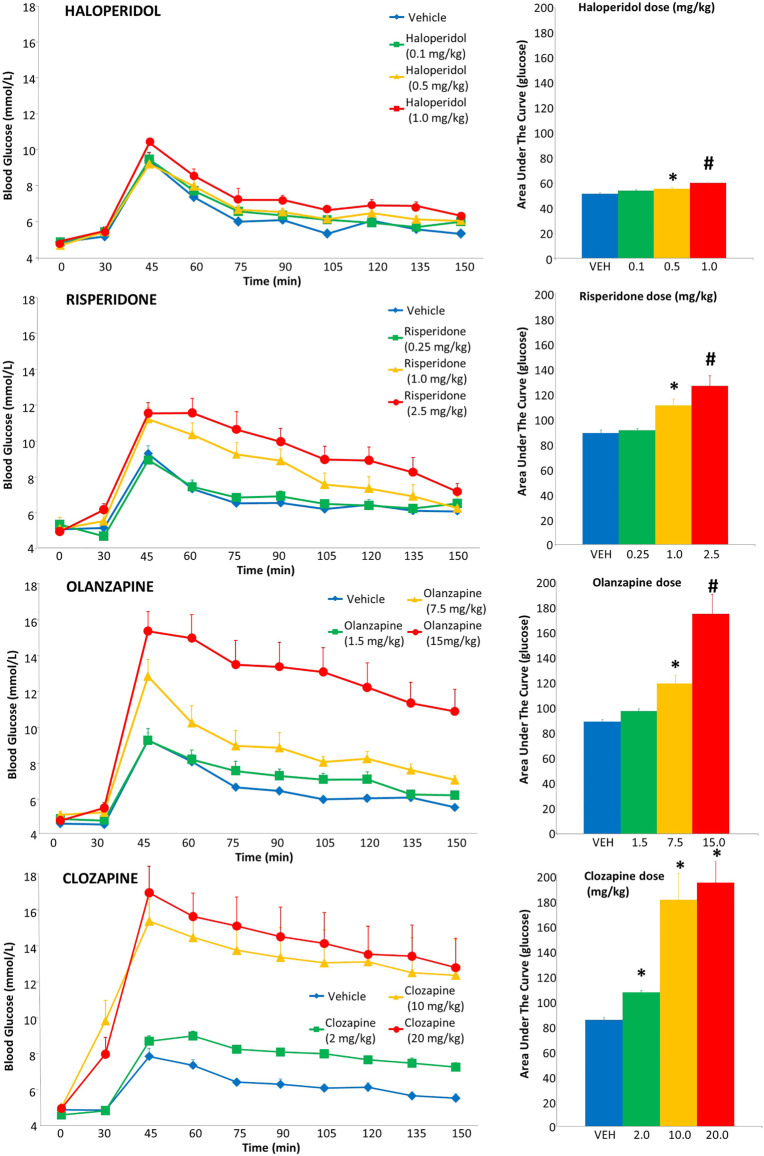
Acute effects of antipsychotics drugs on glucose levels in adult female rats. Animals (*n* = 10–11 per group) received a single injection of vehicle or haloperidol (.1, .5, 1.0 mg/kg, s.c), risperidone (.25, 1.0, 2.5 mg/kg, s.c), olanzapine (1.5, 7.5, 15.0 mg/kg, s.c) or clozapine (2.0, 10.0, 20.0 mg/kg, s.c). Glucose levels were recorded prior to drug treatment in overnight-fasted rats at time = 0, and then 30 min following drug administration (*x*-axis). Immediately following this glucose measurement, all rats were subjected to a glucose tolerance test by receiving an intraperitoneal challenge injection of 1 mg/ml/kg of glucose, and blood glucose levels were measured every 15 min for the next 2 hours. Total cumulative glucose levels for each treatment group are summed as the “area under the curve” during the glucose tolerance test to the right. Values represent group means ± SEM. * statistically significant difference compared to vehicle group, *p* < .05; # statistically significant difference compared to all other groups, *p* < .05.

For haloperidol, there were no group differences in glucose levels prior to the glucose challenge, either before or after drug administration (Figure 2). However, following the glucose injection, there was a significant main effect of drug dose [F_(3,37)_ = 7.02, *p* = .001] and time [F_(8,296)_ = 136.94, *p* < .001]. Post-hoc analysis revealed a dose-dependent increase in glucose levels [measured in area-under-the-curve (AUC)], whereby the .5 and 1.0 mg/kg doses were both significantly greater than vehicle (+8% and +18% respectively), with the latter dose significantly higher than all other groups.

For risperidone, there was no difference in baseline glucose levels (Figure 2), but a group difference following drug treatment prior to the glucose challenge [F_(3,40)_ = 5.67, *p* ≤ .005], evident as higher glucose levels compared to vehicle in the 2.5 mg/kg group. Following the glucose challenge, there was a main effect of drug dose [F_(3,37)_ = 12.08, *p* < .001], time [F_(8,296)_ = 88.74, *p* < .001] and a dose-by-time interaction [F_(24,296)_ = 4.25, *p* < .001]. Follow up with post-hoc tests indicated that the glucose AUC levels were significantly greater in the 1.0 and 2.5 mg/kg doses than both the vehicle (+25% and +42% respectively) and .25 mg/kg dose, with the 2.5 mg/kg dose being significantly greater than all other groups.

With olanzapine, baseline glucose levels did not differ (Figure 2), but there was a main effect of a group difference following drug treatment, pre-glucose challenge [F_(3,40)_ = 5.67, *p* ≤ .005], evident as higher glucose levels compared to vehicle in the 7.5 and 15 mg/kg groups. Following the glucose challenge, there was a main effect of drug dose [F_(3,37)_ = 18.23, *p* < .001], time [F_(8,296)_ = 62.23, *p* < .001] and a dose-by-time interaction [F_(24,296)_ = 4.13, *p* < .001]. Post-hoc tests indicated the glucose AUC levels were significantly greater in the 7.5 and 15 mg/kg doses than the vehicle group (+34% and +96%, respectively), with the 15 mg/kg dose being significantly greater than all other groups.

For clozapine, one rat (10 mg/kg dose) had a seizure and so its IGTT data were excluded. Baseline glucose levels did not differ (Figure 2), but there was a main effect of a group difference following drug treatment pre-glucose challenge [F_(3,39)_ = 9.56, *p* ≤ .001], evident as higher glucose levels compared to vehicle and the 2.5 mg/kg group by both the 10 and 20 mg/kg groups. After the glucose challenge, there was a main effect of drug dose [F_(3,36)_ = 16.68, *p* < .001], time [F_(8,288)_ = 36.24, *p* < .001] and a dose-by-time interaction [F_(24,288)_ = 2.75, *p* < .001]. Follow up with post-hoc tests indicated the glucose AUC levels were significantly greater in the 10 and 20 mg/kg doses than the vehicle group (+92% and +130% respectively) and also greater than the 2 mg/kg dose.

### Plasma Catecholamines

#### Catecholamine Data Were Analyzed Separately for Each Antipsychotic Drug

**FIGURE 3:**
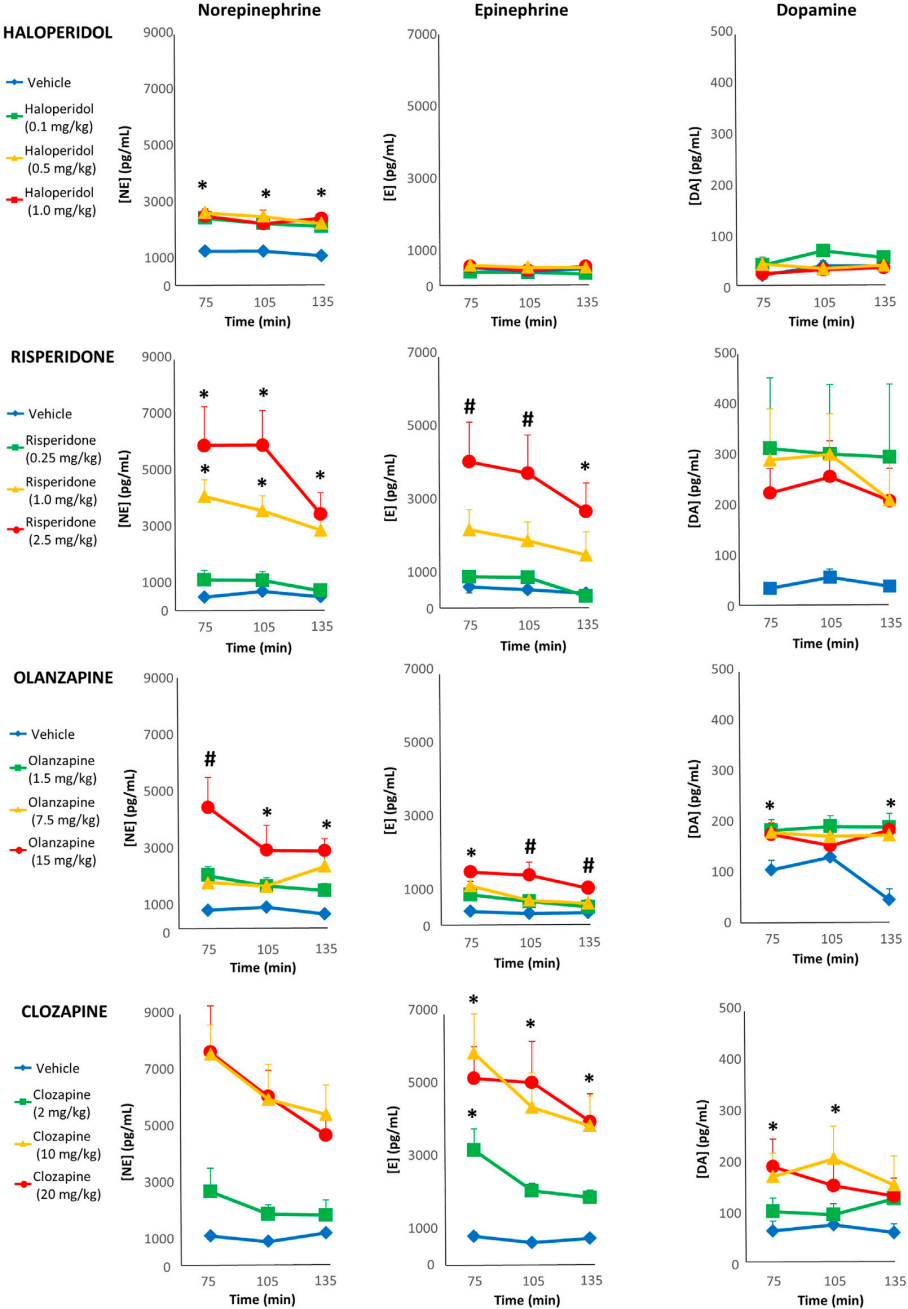
Acute changes in plasma catecholamines after antipsychotic drug treatment in adult female rats. Animals (*n* = 10–11 per group) received a single injection of vehicle or haloperidol (.1, .5, 1.0 mg/kg, s.c), risperidone (.25, 1.0, 2.5 mg/kg, s.c), olanzapine (1.5, 7.5, 15.0 mg/kg, s.c) or clozapine (2.0, 10.0, 20.0 mg/kg, s.c). Separate blood draws were collected 75, 105 and 135 min after drug treatment. Samples were anlayzed by HPLC-ED, and concentrations of norepinephrine (left column), epiphrine (midle column) and dopamine (right column) were determined. Values are expressed in group means ± SEM. * statistically significant difference compared to vehicle group, *p* < .05; # statistically significant difference compared to all other groups, *p* < .05.

For haloperidol, three individual samples (two separate rats, .5 mg/kg group) were spoiled due to an error with the HPLC-ED processing, and so values were replaced with the next observation carried backward/forward as we have conducted previously (Blankers et al., 2010). With norepinephrine, the repeated-measures ANOVA indicated a main effect of dose [F_(3,35)_ = 19.96, *p* < .001]; comparing the AUC levels of norepinephrine, all haloperidol doses showed a significant increase (+92%, +108% and +102% respectively) compared to vehicle (Figure 3). For epinephrine, repeated-measures ANOVA noted no effect of main effect of dose, but a statistically marginal trend for time [F_(2,74)_ = 2.96, *p* = .058]. Exploratory post-hoc tests of the epinephrine AUC showed levels were significantly higher with the 0.5 mg/kg dose (+47%) compared to vehicle, but it was not significant (*p* = .088) for the higher 1.0 mg/kg dose (+39%). With dopamine, there was a non-significant effect of dose [F_(3,37)_ = 2.41, *p* = .082].

With risperidone, all three samples from two rats (.25 mg/kg dose) were spoiled in processing, and so excluded from analysis. For norepinephrine levels, repeated-measures ANOVA reported a main effect of dose [F_(3,35)_ = 17.75, *p* < .001] and time [F_(2,70)_ = 3.96, *p* < .05]. Post-hoc analysis of the norepinephrine AUC noted that both the 1 and 2.5 mg/kg doses caused significantly higher levels compared to vehicle (+549% and +841%, respectively) as well as the .25 mg/kg dose (Figure 3). With epinephrine there was a main effect of dose [F_(3,35)_ = 6.17, *p* < .005] and time [F_(2,70)_ = 8.09, *p* < .005]. Post-hoc analysis of the epinephrine AUC levels noted that they were significantly higher in the 2.5 mg/kg group that vehicle (+598%), but this was only a non-significant trend for the 1 mg/kg group (+267%). For dopamine, there were no significant main effects.

For olanzapine, two individual samples (one rat, vehicle group) were spoiled due to an error with the HPLC-ED processing, and so values were replaced with next observation carried backward/forward. The repeated-measures ANOVA of norepinephrine levels detected a main effect of drug dose [F_(3,37)_ = 15.84, *p* < .001]; comparing the AUC levels of norepinephrine, all three dose of olanzapine increased levels significantly compared to vehicle (+121%, +147% and +337%, respectively) (Figure 3), while the 15 mg/kg exhibited higher levels than all other groups. For epinephrine, there were main effects of dose [F_(3,37)_ = 15.17, *p* < .001] and time [F_(2,74)_ = 7.07, *p* < .005]. Post-hoc analysis of epinephrine AUC levels indicated that all doses of olanzapine were significantly higher than the vehicle group (+86%, +121% and +260%, respectively), and the 15 mg/kg exhibited higher levels than all other groups. Analysis of dopamine levels revealed a main effect of dose [F_(3,37)_ = 5.29, *p* < .005] and dose-by-time interaction [F_(6,74)_ = 2.52, *p* < .05]. Follow-up post-hoc tests of the dopamine AUC reported that all three doses of olanzapine elevated levels significantly compared to the vehicle group (+104%, +89% and +85%, respectively).

With clozapine, one sample of norepinephrine (10 mg/kg group) was identified by SPSS as an extreme outlier (>5× the interquartile range) and so excluded from analysis. The repeated-measures ANOVA of norepinephrine levels indicated main effects of dose [F_(3,36)_ = 17.04, *p* < .001] and time [F_(2,72)_ = 4.63, *p* < .05]. The post-hoc tests of the norepinephrine AUC noted that all doses were significantly higher than vehicle (+101%, +499% and +482%, respectively), while the 10 and 20 mg/kg were also higher than the 2 mg/kg dose (Figure 3). A similar pattern emerged for epinephrine, with main effects of dose [F_(3,36)_ = 10.54, *p* < .001] and time [F_(2,72)_ = 6.94, *p* < .005], where post-hoc tests of the epinephrine AUC observed that both the 10 and 20 mg/kg doses exhibited significantly greater levels (+575% and +581%) than vehicle and the 2 mg/kg dose. The repeated-measures ANOVA revealed an effect of clozapine dose on dopamine levels [F_(3,36)_ = 3.20, *p* < .05], and post-hoc analysis of the dopamine AUC showed that both the 10 and 20 mg/kg doses had significantly greater levels than the vehicle group (+162% and +135%, respectively).

### Relationship Between Catecholamine Levels and Glucose Intolerance

To examine the relationship between the catecholamines and glucose intolerance for the four antipsychotic drugs, individual glucose values were correlated with catecholamine levels at the three timepoints (*t* = 75, 105 and 135 min) when they were concurrently measured. Additionally, we also correlated the AUC of glucose levels during the entire IGTT with the AUC of catecholamine levels for the three timepoints measured (Table 1).

**TABLE 1:** Associations between glucose intolerance and plasma catecholamine levels in adult female rats.

Glucose	NE	NE	NE	NE	E	E	E	E	DA	DA	DA	DA
	*t* = 75	*t* = 105	*t* = 135	AUC	*t* = 75	*t* = 105	*t* = 135	AUC	*t* = 75	*t* = 105	*t* = 135	AUC
Haloperidol												
*t* = 75	−.068				.188				−.068			
	NS				NS				NS			
*t* = 105		.364				.443				−.164		
		*p* < .05				*p* < .005				NS		
*t* = 135			.419				.474				−.116	
			*p* < .01				*p* < .005				NS	
AUC				.311				.446				−.243
				*p* < .05				*p* < .005				NS
Risperidone												
*t* = 75	.617				.553				.169			
	*p* < .001				*p* < .001				NS			
*t* = 105		.603				.691				.322		
		*p* < .001				*p* < .001				*p* < .05		
*t* = 135			.670				.659				.157	
			*p* < .001				*p* < .001				NS	
AUC				.836				.805				.294
				*p* < .001				*p* < .001				*p* = .07
Olanzapine												
*t* = 75	.436				.591				−.012			
	*p* < .005				*p* < .001				NS			
*t* = 105		.394				.448				.045		
		*p* < .05				*p* < .005				NS		
*t* = 135			.485				.651				.251	
			*p* = .001				*p* < .001				NS	
AUC				.697				.668				.159
				*p* < .001				*p* < .001				NS
Clozapine												
*t* = 75	.538				.568				.455			
	*p* < .001				*p* < .001				*p* < .005			
*t* = 105		.528				.540				.451		
		*p* < .001				*p* < .001				*p* < .005		
*t* = 135			.668				.685				.615	
			*p* < .001				*p* < .001				*p* < .001	
AUC				.656				.688				.602
				*p* < .001				*p* < .001				*p* < .001

Animals (*n* = 10–11 per group) received a single injection of vehicle or haloperidol (.1, .5, 1.0 mg/kg, s.c), risperidone (.25, 1.0, 2.5 mg/kg, s.c), olanzapine (1.5, 7.5, 15.0 mg/kg, s.c) or clozapine (2.0, 10.0, 20.0 mg/kg, s.c). All animals completed a glucose tolerance test, from t = 30–150 min after drug treatment. Peripheral catecholamine levels were also measured 75, 105 and 135 min after drug treatment. The table reports the Pearson correlation coefficients and statistical significance between blood glucose levels and plasma norepinephrine (NE), epinephrine (E) and dopamine (DA) levels at *t* = 7, 105 and 135 min. Total blood glucose levels during the glucose tolerance test are noted as the area-under-the-curve (AUC) and correlated with total catecholamine levels for all three time points, also represented as the AUC. Data for each antipsychotic drug were analyzed separately; NS, non-significant.

For haloperidol, there were moderate, significant correlations (*r* = 0.311–.474) between glucose levels and norepinephrine/epinephrine at *t* = 105 and *t* = 135 min, as well as when timepoints were aggregated with the AUCs. There was no correlation between glucose and dopamine levels. For risperidone, there were strong, highly significant correlations (*r* = .553–.836) between glucose levels and norepinephrine/epinephrine at all time points, as well as with the AUCs. With dopamine, there was a significant correlation (*r* = .322) at *t* = 105 min, and a non-significant trend with the AUCs (*r* = .294, *p* = .07). For olanzapine, again, there were mostly strong, significant correlations (*r* = .394–.697) between glucose levels and norepinephrine/epinephrine at all time points and AUCs, although no significant association between glucose and dopamine levels. Finally, with clozapine, there were strong, highly significant correlations (*r* = .451–.688) between glucose levels and all timepoints and AUCs, for all catecholamines—including dopamine.

The mediation analyses confirmed that for most of the stronger correlations, the catecholamine levels served as significant (*p* < .05) mediators of the effect of drug dose on glucose intolerance. Significant test effects, whereby the AUC of catecholamine levels acted as an intervening variable for drug dose on the AUC in the IGTT, were noted for haloperidol (norepinephrine AUC, Z-score = 3.62), risperidone (norepinephrine AUC, Z-score = 6.86; epinephrine AUC, Z-score = 4.41), olanzapine (norepinephrine AUC, Z-score = 2.12; epinephrine AUC = non-significant trend (*p* = .08) and clozapine (norepinephrine AUC, Z-score = 2.20; epinephrine AUC, Z-score = 2.70; dopamine AUC, Z-score = 2.01). Interestingly, for olanzapine, mediator effects were stronger when dose was entered into the equations as an ordinal, rather than continuous, variable reflecting possible non-linear effects.

## Discussion

The goal of the current study was to determine if treatment with antipsychotic drugs resulted in elevation of peripheral catecholamines concurrently with metabolic dysregulation, measured by glucose intolerance. Our overall findings strongly confirmed this hypothesis. Consistent with prior preclinical observations, the four different antipsychotic drugs used in the present study all caused potent glucose intolerance, and this was largely dose-dependent. Reflecting the pharmacological validity of the model, the effects on glucose intolerance paralleled those seen in the clinic, whereby the smallest metabolic effects were noted with haloperidol, and the largest with clozapine. In the same animals, blood that was collected at three separate timepoints during the GTT confirmed large increases in peripheral catecholamines, which was statistically significant for the three second-generation drugs at the higher doses. Importantly, there were strong, highly significant associations between levels of norepinephrine/epinephrine and elevated plasma glucose levels for most of the antipsychotics, as well as for dopamine with clozapine. Mediation analyses indicated that most of these correlations represented intervening effects between the drug treatment and glucose intolerance, whereby a large proportion of the effects of drug dose on glucose intolerance were directly mediated through the increases in catecholamine concentrations.

These findings therefore provide additional evidence that peripheral catecholamines may play a key role in the acute metabolic effects of antipsychotic drugs. The metabolic side-effects of antipsychotic drugs in clinical populations are now well established (Allison et al., 1999; McEvoy et al., 2005; Newcomer, 2007; Kessing et al., 2010; Mitchell et al., 2013; Kim et al., 2017; Tumiel et al., 2019; Yuen et al., 2021a), and animal studies have faithfully modelled these many of these effects, using comparable techniques that are commonly used in humans, including the GTT (Monzillo and Hamdy, 2003). We and many other research groups (Smith et al., 2008; Muller et al., 2010; Boyda et al., 2013a; Boyda et al., 2014; Evers et al., 2017; Lord et al., 2017; Stefanidis et al., 2017; Oxenkrug and Summergrad, 2020; Sylvester et al., 2020; Shamshoum et al., 2021) routinely observe that antipsychotics generate acute glucose intolerance of an equivalent magnitude to that noted in human studies (Albaugh et al., 2011) without a change in factors such as food intake or obesity, indicating a direct effect of the drugs on glucose dysregulation. Furthermore, previous experiments in our laboratory have reported that these effects on glucose intolerance remained stable with repeated, chronic treatment over months (Boyda et al., 2012; Boyda et al., 2014), comparable to the human condition. The drug-specific degree of glucose intolerance exhibited by the current four antipsychotic drugs is homologous with the degree of metabolic dysregulation noted in patients, which typically follows the sequence: clozapine > olanzapine ≥ risperidone > haloperidol (Tandon and Halbreich, 2003; Newcomer, 2005). Our observations noted increased AUC glucose levels of +130%, +96%, +42% and +18% with the highest doses of each of these drugs, respectively. The present findings therefore suggest that these acute metabolic effects may provide a meaningfully predictive model for further understanding the underlying physiology.

In parallel to glucose intolerance, most doses of drugs also caused large increases in the peripheral catecholamines, with the greatest increases (both in absolute and relative terms) in norepinephrine, followed by epinephrine and then dopamine. The pattern of elevated catecholamines was slightly different to that of the IGTT, with the largest changes caused by clozapine, followed by risperidone (rather than olanzapine), olanzapine and then haloperidol. However, it should be noted that the increases in norepinephrine were large even for haloperidol, whereby all doses approximately doubled norepinephrine levels. These effects of the antipsychotics on peripheral catecholamine levels recapitulate the findings from our recent study (Boyda et al., 2020), where we measured catecholamine levels with the same drugs, but with fewer doses and without any measure of metabolic dysregulation. This suggests that these effects are stable and reliable, and also consistent with a limited number of other studies in the preclinical literature which have observed the effects of individual antipsychotics on specific catecholamines, including one which noted that intravenous treatment with the first-generation drug chlorpromazine caused an increase of both norepinephrine and epinephrine (Sato et al., 1988). Meanwhile, indirect evidence for increased catecholamines was evident in a study where hyperglycemia, caused by treatment with clozapine and chlorpromazine, was mitigated by co-treatment with the ganglionic blocker hexamethonium, which presumably worked by preventing increases in peripheral norepinephrine and epinephrine (Savoy et al., 2010) - although these were not actually measured in the study. Human clinical findings also indicate that both norepinephrine and epinephrine are elevated by antipsychotic drugs with higher metabolic liability (Green et al., 1993; Schulz et al., 1996; Brown et al., 1997; Elman et al., 1999). For instance, two well-controlled studies observed that levels of peripheral norepinephrine were three times greater in patients treated with clozapine than either of the first-generation drugs fluphenazine or haloperidol (Brown et al., 1997; Elman et al., 1999). However, to our knowledge, clinical studies have yet to determine a link between peripheral catecholamine levels and metabolic dysregulation.

It is therefore apposite that in the present study, there were strong and highly significant correlations between glucose levels and norepinephrine/epinephrine concentrations for all four antipsychotics. While associations clearly do not determine causality, the consistent and robust correlations for all four drugs between glucose intolerance and norepinephrine/epinephrine levels—as well as the results of the mediation analyses—provide data to suggest that further study of the link between these two physiological pathways is warranted. Both norepinephrine and epinephrine serve as hormones that are highly active in the regulation of many metabolic processes, and are therefore strong candidates—*a priori*—to examine with regards to the metabolic side-effects of antipsychotics. Pertaining to glycemic control, both catecholamines play an essential role in glycogenolysis, hepatic gluconeogenesis, glucagon secretion and insulin release (Barth et al., 2007; Boyda et al., 2013b). Elevated concentrations of both norepinephrine and epinephrine might therefore be expected to contribute in manifold ways to excessively elevated glucose levels, and will require future study—although some potential candidate substrates of these catecholamines, such as glucagon, are already being confirmed as presumed downstream mediators of antipsychotic-induced glucose intolerance (Medak et al., 2019; Shamshoum et al., 2021). Physiologically, the relative importance of each of the individual catecholamines towards the drug-induced glucose dysregulation will require more targeted evaluation, involving both pharmacological and anatomical techniques [such as denervation of the pancreas (Nilsson et al., 2001)]. With regards to the former, in a recent study we sought to understand which of the adrenoceptors may be responsible for mediating the effects of putative increases in peripheral catecholamines on whole-body insulin resistance (Yuen et al., 2021b) following treatment with clozapine. Using selective antagonists at α1 (prazosin), α2 (idazoxan), β1 (atenolol) and β2 (butoxamine) adrenoceptors, we observed that both atenolol and butoxamine could significantly reverse clozapine-induced insulin resistance. Additionally, the ganglionic blocker mecamylamine was able to partially reverse the insulin resistance caused by treatment with clozapine. Mecamylamine is a potent non-selective antagonist at nicotinic receptors, which blocks the effects of acetylcholine released by preganglionic nerve fibers, thereby preventing downstream release of norepinephrine from post-ganglionic fibers and epinephrine from the adrenal medulla. Less well understood is the role of peripheral dopamine as a potential mediator of metabolic side-effects. In the body’s periphery, dopamine can be released from three main sources, including neuronal fibers, the adrenal medulla, and neuroendocrine cells (Pearse, 1969). It exerts its hormonal effects across a diverse range of substrates. There is considerable evidence to show that peripheral dopamine regulates body weight and glucose homeostasis via insulin release (Rubí and Maechler, 2010). Studies have demonstrated that dopamine directly inhibits the secretory response of β-cells in the pancreas, and dopamine D2 receptors are expressed in both rodent and human islets, where the receptors regulate inhibition of the secretory response (Rubí et al., 2005).

While the present study is hopefully informative, and provides additional novel evidence to support a key role for the peripheral catecholamines on antipsychotic metabolic side-effects, there are a number of limitations. Firstly, we studied four antipsychotic drugs, but there are a considerably greater number of antipsychotic drugs than this in clinical use. While the full range of metabolic liability was represented with the current drugs, spanning from haloperidol to clozapine (Newcomer, 2005), it will be important to determine how other antipsychotics with known metabolic side-effects (Barr et al., 2006; Houseknecht et al., 2007) work in this model. It will be of particular interest to assess the impact of third generation antipsychotic drugs, which exert partial agonist activity at the dopamine D2 receptor—but also serve as functional antagonists when the endogenous ligand is at a higher concentration (Mailman and Murthy, 2010). Given that peripheral dopamine can regulate insulin release in the pancreas (Rubí and Maechler, 2010), the impact of this partial agonism/antagonism under basal and stimulated levels of dopamine before and following antipsychotic treatment will need further study. It will also be interesting to study a drug such as aripiprazole in combination with clozapine, which has been shown to reduce the metabolic side-effects of the latter (Fan et al., 2013). Whether aripiprazole’s partial agonist effect at D2 receptors at peripheral sites, such as the pancreas, might serve to counter significant elevations in catecholamines (including dopamine) caused by clozapine will be feasible to study. A second major limitation of the present study is, the doses of drugs that were used in the present study may be considered high, in relation to doses needed to achieve occupancy of mesolimbic dopamine receptors at the same degree as observed with antipsychotic effects in humans (Kapur et al., 2003). This could theoretically call into question the external validity and translational value of the model. While this remains uncertain at present, there is considerable evidence that the physiological substrates and pathways that mediate the therapeutic and metabolic side-effects of antipsychotic drugs are different (Reynolds, 2012), and so may not be equally as sensitive to drug effects in different species. It may be that in rats, comparably higher doses are needed to observe metabolic effects of an equivalent magnitude to those seen in humans. For example, Albaugh et al. (2011) acutely administered a therapeutic dose of olanzapine to healthy human volunteers and observed an increase in glucose intolerance (measured by the AUC) of 42%. In our laboratory, acute treatment with olanzapine in rats requires a dose of at least 10 mg/kg to observe a comparable increase in glucose intolerance of 39% (Wu et al., 2014); this dose is notably greater than that needed to achieve species-equivalent “therapeutic” D2 receptor occupancy. Further study will therefore be necessary to determine the relationship between drug dosing for side-effects vs efficacy in rodent models to better understand the relevance to the human condition. A third important limitation of our study is that we only used female rats. The preclinical literature on antipsychotic drug-induced metabolic side-effects reveals a complex pattern when comparing between males and females, in which effects vary depending on both the specific drug and metabolic parameter being studied (see Castellani et al. (2019) for a comprehensive review). With regards to glucose intolerance, it has been claimed that female rats exhibit less reliable acute glucose dysregulation than males (Castellani et al., 2019). For the specific purposes of studying acute mechanisms associated with drug effects, the use of one sex alone may be justified in the earlier stages of exploratory research. However, it will be important in the future to compare the effects of antipsychotic drugs on peripheral catecholamine levels and both males and females to increase the generalizability to the clinical population. A fourth limitation of the present study is the administration of antipsychotic drugs acutely rather than chronically, as per the patient population. It is entirely possible that the current increases in peripheral catecholamines may diminish or disappear with repeated treatment. In our laboratory, we have observed consistent effects of olanzapine with chronic treatment over 9–10 weeks (Boyda et al., 2012; Boyda et al., 2014), in a manner whereby the magnitude of glucose tolerance remains stable and does not increase or diminish after weeks or months. It will therefore be important to determine if peripheral catecholamines follow this same trajectory. Interestingly, we did observe that intermittent treatment (vs continuous treatment) was associated with a “sensitization” of metabolic effects (Boyda et al., 2012), which is consistent with catecholamines’ role in central sensitization (Featherstone et al., 2007). Clinical studies that have noted elevated peripheral epinephrine and norepinephrine in patients treated with second generation antipsychotics did so in individuals who had received chronic treatment (Brown et al., 1997; Spivak et al., 1998; Elman et al., 2002).

## Conclusion

In conclusion, the current experiments provide further evidence for the potential role of peripheral catecholamines in the metabolic side-effects of antipsychotic drugs. Acute treatment with four different antipsychotics of varying metabolic liability in humans induced similar changes in glucose intolerance, and these metabolic changes were strongly associated with increases in peripheral concentrations of norepinephrine and epinephrine. These findings indicate that further research into the links between glucose dysregulation and elevated catecholamines may provide a productive avenue for future research, while acute measurement of peripheral catecholamines may have predictive potential for antipsychotic-induced metabolic dysregulation, when assessed using procedures such as the GTT.

## Data Availability

The raw data supporting the conclusion of this article will be made available by the authors, without undue reservation.
